# Ascites fluid calprotectin level is highly accurate in diagnosing spontaneous bacterial peritonitis: a preliminary proof of concept prospective study

**DOI:** 10.1007/s10238-023-01257-0

**Published:** 2024-01-28

**Authors:** Wisam Sbeit, Basheer Maamoun, Subhi Azzam, Amir Shahin, Michal Carmiel-Haggai, Tawfik Khoury

**Affiliations:** 1 Department of Gastroenterology, Galilee Medical Center, Nahariya, Israel; 2 Azrieli Faculty of Medicine, Bar-Ilan University, Safed, 1311502 Israel; 3 Liver Unit, Galilee Medical Center, Nahariya, Israel

**Keywords:** Cirrhosis, Ascites, Calprotectin, Yield, Stage

## Abstract

Ascites is the most common complication of liver cirrhosis. Spontaneous bacterial peritonitis (SBP) is a common complication of ascites. The diagnosis is made by an ascitic fluid polymorphonuclear (PMN) cell count of ≥ 250/mm^3^. However, no other diagnostic test is present for the diagnosis of SBP. The aim of the study present study is to assess the diagnostic yield of ascitic calprotectin in SBP, and to explore whether it can predict disease stage. We performed a single center proof-of-concept prospective study including all patients with cirrhosis and ascites who underwent paracentesis. Overall, 31 patients were included in the study. Eight patients had SBP vs. 23 patients without SBP. Ascitic calprotectin level was 77.4 ± 86.5 μg/mL in the SBP group, as compared to 16.1 ± 5.6 μg/mL in the non-SBP group (*P* = 0.001). An ascitic calprotectin cut-off value of > 21 μg/mL was associated with sensitivity and specificity of 85.7% and 89.5%, respectively, with ROC of 0.947 (95% CI 0.783 to 0.997, *P* < 0.0001). Notably, ascitic calprotectin did not had a prognostic value in cirrhosis stage and prognosis. Ascitic calprotectin was highly accurate in the diagnosis of SBP. It can be a serve as adjunct for indefinite cases of SBP.

## Introduction

Liver cirrhosis is the final common irreversible stage of chronic liver diseases of variable etiologies. Cirrhotic patients are susceptible to various life threatening complications, known as decompensated cirrhosis including variceal hemorrhage, ascites, spontaneous bacterial peritonitis, hepatic encephalopathy, hepatorenal syndrome, hepatopulmonary syndrome and hepatocellular carcinoma, developing in 58% of patients 10 years after diagnosis [1]. Ascites represents the most common complications of cirrhosis [2]. Spontaneous bacterial peritonitis (SBP) is infection of the ascetic fluid developing in end stage cirrhosis in the absence of an evident surgically treatable intra-abdominal source of infection [3]. SBP is almost always monomicrobial and defined by a positive bacterial culture and an ascitic fluid polymorphonuclear (PMN) cell count of ≥ 250/mm^3^ [3]. Neutrophils are the principal effector cells that characterize acute inflammation in response to a wide range of factors. When they migrate to a site of chemoattraction, a chain of event start out that leads inter alia to their disruption and release of their cytosolic granules including calprotectin [4]. Calprotectin is a 36.5 kd zinc and calcium binding protein of the S100 family, that is mostly derived from neutrophils and monocytes [5, 6]. As it can be detected in tissue samples, body fluids, and stools it represents an attractive marker of neutrophil activity and plays a role in diagnosis, monitoring and determining prognosis of different gastrointestinal diseases [6]. Importantly, fecal calprotectin is highly resistant to degradation by intestinal pancreatic secretions, intestinal proteases, and bacterial degradation, making it an optimal biomarker of inflammation [4]. Due to the fact that there is a significant correlation between fecal calprotectin level and other measures of acute inflammation and severity in inflammatory bowel diseases [7, 8]. As neutrophils are the principal effector cells in SBP, and as ascitic fluid calprotectin is highly resistant to bacterial degradation, its level may represent a precise measure of inflammation. The aims of our study are to investigate the diagnostic role of ascites fluid calprotectin in SBP, and to investigate whether ascites fluid calprotectin has prognostic prediction role in advanced decompensated cirrhosis and its relation to disease severity as assessed by Child–Pugh Score and Model for End-Stage Liver Disease (MELD) score.

## Materials and methods

We conducted a prospective study, recruiting cirrhotic patients with ascites treated in the outpatient clinic or hospitalized at Galilee Medical Center. All those patients were considered potentially eligible for enrollment in the study after obtaining informed consent. Patients who are planned to undergo ascitic fluid paracentesis as decided by the treating physician regardless to this research were included in our study. Fluid was collected for biochemistry, including protein, albumin, glucose; microscopy for red cell count and white cell count including neutrophils; culture and calprotectin level. In addition, blood serum was tested for complete blood count, glucose, blood urea nitrogen, creatinine, protein, albumin, aspartate aminotransferase (AST), alanine aminotransferase (ALT), alkaline phosphatase (ALP), bilirubin and prothrombin time. Additionally, collected data include demographic variables (age, gender), hospitalization vs. outpatient setting, liver disease etiology and background diseases. The diagnosis of liver cirrhosis in our cohort was confirmed by computed tomography showing the classical signs of cirrhosis including irregular liver contour and liver lobes atrophy, and FibroScan® elastography. Calprotectin level was assessed by enzyme-linked immunosorbent assay (ELISA) in ascites fluid. The kit was bought from CALPRO AS, Lysaker, Norway. ELISA kits usually use 80 to 100 cells for measurement, including for quality controls, and all samples were analyzed in duplicate.

**Inclusion criteria**: All patients older than 18 years suffering from liver cirrhosis with ascites who are planned for paracentesis regardless to this research, were offered to participate in the study, and those who gave their informed consent were consequently recruited.

**Exclusion criteria**: Patients with ascites not related to liver cirrhosis and portal hypertension.

### Statistical analysis

Patient characteristics were presented as arithmetic means (± standard deviation (SD)) or range for continuous variables, or as frequencies (percentages) for categorical variables. The study cohort was divided into patients with and without SBP. Descriptive statistics after the was used for the comparison of the continuous variables using the student’s t-test, and comparison of the categorial variables was performed by the Fisher's exact test. *P* values of less than 0.05 were considered statistically significant. Statistical analysis was performed with commercial software. All analyses were performed using IBM SPSS Statistics, version 25.

## Results

### Demographics and baseline characteristics

Overall, 31 patients were included during the study period. Among them, 8 patients were diagnosed with SBP according to the current guidelines (> 250 PMN/mm) (group A), as all patients were hospitalized, compared to 23 patients who did not have SBP (group B). The average age in group A was 69.3 ± 12.9 years in group A, vs. 65.8 ± 10 years in group B (*P* = 0.2). The male to female ratio was 1 (12.5%)/7 (87.5%) in group A, as compared to 13 (56.5%)/10 (43.5%) in group B (*P* = 0.01). None of the patients in group A was a smoker, as compared to 5 patients (21.7%) in group B (*P* = 0.08). Table 1 demonstrates demographics and background diseases. The most common cause of liver cirrhosis was non-alcoholic steatohepatitis (NASH) in 75% and 78.3% in groups A and B, respectively (*P* = 0.8). Moreover, the indication of paracentesis was suspected SBP in 100% in group A, and 78.3% in group B. The vital signs and the clinical scores (MELD and Child–Pugh) at the time of the paracentesis are demonstrated at Table 2.

**Table 1:** Demographics and baseline characteristics

	SBP group	Non-SBP group	*P* value
Number	8	23	–
Age, mean ± SD (years)	69.3 ± 12.9	65.8 ± 10	0.2
Gender, N (%)			
Male	1 (12.5)	13 (56.5)	0.05
Female	7 (87.5)	10 (43.5)	
Weight (Kg)	78.2 ± 19.5	74.5 ± 16.4	0.3
Height (cm)	154.3 ± 6.7	164.2 ± 8.6	0.008
Body mass index	31.7 ± 4.9	27.7 ± 5.2	0.04
Smoking, N (%)	0	5 (21.7)	0.08
Congestive heart failure, N (%)	0	8 (34.8)	0.1
Hypertension, N (%)	5 (62.5)	8 (34.8)	0.2
Diabetes mellitus, N (%)	6 (75)	16 (69.6)	0.7
Hyperlipidemia, N (%)	2 (25)	10 (43.5)	0.3
Non-alcoholic fatty liver disease, N (%)	5 (62.5)	8 (34.8)	0.2
Hepatitis B virus, N (%)	1 (12.5)	0	0.2
Hepatitis C virus, N (%)	0	3 (13)	0.5
Atrial fibrillation, N (%)	0	8 (34.8)	0.1
Obstructive lung disease, N (%)	0	2 (8.7)	0.6
Renal failure, N (%)	1 (12.5)	3 (13)	0.9

**Table 2:** Clinical characteristics of the study cohort

	SBP group	Non-SBP group	*P* value
Ascites cause, N (%)			
NASH	6 (75)	18 (78.3)	0.8
Hepatitis B	1 (12.5)	1 (4.3)	0.4
Hepatitis C	1 (12.5)	5 (21.7)	0.6
Alcoholic hepatitis	0	2 (8.7)	0.6
Congestive hepatopathy	0	1 (4.3)	0.9
Indication for paracentesis, N (%)			
Suspected SBP	8 (100)	18 (78.3)	–
Tense ascites	5 (62.5)	18 (78.3)	
Systolic blood pressure (mmHg), average ± SD	128.6 ± 20.3	115.6 ± 14.8	0.03
Diastolic blood pressure, average ± SD	71.6 ± 6.7	68.2 ± 9.4	0.1
Heart rate, average ± SD	87.1 ± 14	83.1 ± 16.7	0.2
Respiratory rate (breath/minute), average ± SD	16.1 ± 1.3	16.1 ± 2	0.4
Temperature, average ± SD	36.9 ± 0.33	36.7 ± 0.4	0.1
MELD score, average ± SD	11.6 ± 4.1	16.1 ± 7.3	0.06
Child–Pugh score, average ± SD	7.9 ± 1.3	9 ± 1.4	0.03

### Laboratory findings

**Fig. 1:**
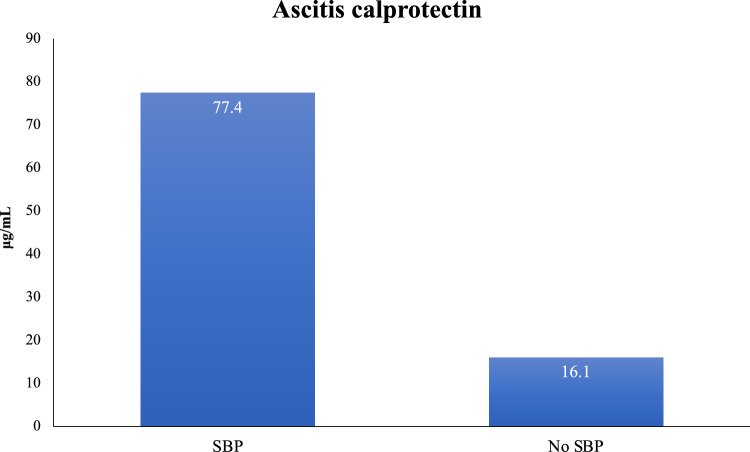
Demonstrates the level of ascitic calprotectin among patients with and without SBP

Table 3 demonstrates the serum laboratory findings of the study cohort. The level of leukocytes was significantly higher in group A, as compared to group B (8.8 ± 3.7 vs. 6 ± 2.7, *P* = 0.01). However, there was no difference in the rest of the serum laboratory findings. Notably, the serum albumin level was not different (3.1 g/l ± 0.8 vs. 2.8 g/l ± 0.6) for groups A and B, respectively (*P* = 0.1) (Table 3). The level of ascitic leukocytes and absolute neutrophils were expectedly significantly higher in group A, as compared to group B (3947.3 ± 5272.8 and 2837.6 ± 4961.5, vs. 239.8 ± 166.4 and 53.9 ± 43.7), *P* = 0.0008 and *P* = 0.004, respectively (Table 4). Interestingly, the level of ascitic calprotectin was significantly higher in group A as compared to group B (77.4 ± 86.5 μg/mL (18.6–500) vs. 16.1 ± 5.6 μg/mL (8.8–32.5), *P* = 0.001) (Fig. 1). The average time took to perform the ascitic calprotectin level was 2 h, vs. 15 min for leukocyte count determination. The cost of complete ascitic fluid count was 10 vs. 8 Euros for calprotectin level assessment. Performing receiver operating characteristic curve (ROC) analysis showed that an ascitic calprotectin cut-off value of > 21 μg/mL was associated with sensitivity of 85.7%, specificity of 89.5%, positive likelihood ratio of 8.1, with ROC of 0.947 (95% CI 0.783 to 0.997, *P* < 0.0001) for the diagnosis of SBP.

**Table 3:** Demonstrates serum laboratory findings

	SBP group	Non-SBP group	*P* value
Hemoglobin (g/dl), average ± SD	9.1 ± 1	9.4 ± 1.7	0.3
Leukocytes (*10e3/ul), average ± SD	8.8 ± 3.7	6 ± 2.7	0.01
Glucose (mg/dl), average ± SD	120 ± 27.5	120.9 ± 33.8	0.4
Blood urea nitrogen (mg/dl), average ± SD	33.6 ± 17.7	42.6 ± 42.5	0.3
Creatinine (mg/dl), average ± SD	1.1 ± 0.2	1.7 ± 1.3	0.1
Total protein (g/dl), average ± SD	6.3 ± 0.4	9 ± 13.9	0.3
Albumin (g/dl), average ± SD	3.1 ± 0.8	2.8 ± 0.6	0.1
ALT (u/l), average ± SD	25 ± 28.7	53.2 ± 135	0.2
ALK (u/l), average ± SD	102.1 ± 58.2	239 ± 292	0.1
GGT (u/l), average ± SD	89.3 ± 74.1	244.4 ± 246.7	0.1
Total bilirubin (g/dl), average ± SD	1 ± 1.2	2.2 ± 3.8	0.2
INR, average ± SD	1.3 ± 0.2	1.6 ± 0.7	0.09

**Table 4:** Demonstrates the ascitic laboratory findings

	SBP group	Non-SBP group	*P* value
Total protein, average ± SD	2.2 ± 1.4	2.3 ± 1.4	0.4
Albumin, average ± SD	1.3 ± 08	1.2 ± 0.7	0.3
Glucose, average ± SD	143.5 ± 81.8	168 ± 77.7	0.2
Leukocytes, average ± SD	3947.3 ± 5272.8	239.8 ± 166.4	0.0008
Absolute neutrophil, average ± SD	2837.6 ± 4961.5	53.9 ± 43.7	0.004

### Comparison of ascites calprotectin in early vs. advanced liver cirrhosis

Among patients who had a mild disease stage as defined by MELD score < 11 (10 patients), the level of ascitic calprotectin was 42.9 ± 67.2, as compared to 18.9 ± 10.2 among the 21 patients who had more advanced liver disease as defined by MELD score of ≥ 11 (*P* = 0.09). In those patients, the rate of SBP was not statistically different between the groups (40% for the MELD score < 11, vs. 20% for MELD score of ≥ 11, *P* = 0.06). Classifying the patients according to the Child–Pugh score, comparing Child–Pugh A and B (mild to moderate disease, 21 patients), vs. Child–Pugh C (severe advanced disease, 10 patients), the ascitic calprotectin level was not different between the two groups (31.2 ± 52.5 for Child–Pugh A and B, and 23.3 ± 12.9 for Child–Pugh C, *P* = 0.3). Again, in those patients, the rate of SBP was similar in both groups (28% for Child–Pugh A and B, and 20% for Child–Pugh C, *P* = 0.3) (Table 5). Therefore, pointing to the absence of a potential prognostic value of ascitic calprotectin in advanced decompensated cirrhosis.

**Table 5:** Ascitic calprotectin level according to disease stage

	Calprotectin level	*P* value
MELD score		
< 11	42.9 ± 67.2	0.09
≥ 11	18.9 ± 10.2	
Child–Pugh score		
A–B	31.2 ± 52.5	0.3
C	23.3 ± 12.9	

## Discussion

The current study aimed to explore the diagnostic role of ascites fluid calprotectin in SBP and the level of ascites fluid calprotectin as prognostic prediction role in advanced decompensated cirrhosis. In this study we showed that ascites fluid calprotectin was significantly higher in SBP group compared to non SBP group as diagnosed by ascetic fluid PMN > 250 (77.4 ± 86.5 vs. 16.1 ± 5.6 respectively, (*P* = 0.001), with a cut-off level of > 21 of > 21 μg/mL was associated with an excellent diagnostic performance for SBP, with ROC of 0.947. Similar results were shown previously in several studies [9–11]. A recent meta-analysis by Hadjivasilis et al. demonstrated that Ascitic calprotectin appears to be an excellent alternative to PMN leucocyte count of ≥ 250 cells/mm^3^ for the diagnosis of SBP with much faster time to diagnosis. Owing to its substantially high negative predictive value, the test can accurately exclude SBP avoiding unnecessary antibiotics in suspected patients [12]. Ascitic fluid calprotectin in cirrhotic patients have a good sensitivity and specificity for SBP. Some of them used ELISA [13–16], while others used point-of-care testing (POCT) [17, 18]. Ascitic fluid calprotectin reliably predicts polymorphonuclear leukocytes (PMNs) count > 250/mm^3^, which may provide a useful marker for the diagnosis of SBP, especially with a readily available bedside testing device [15]. We also found that the level of ascitic calprotectin was significantly higher in patient diagnosed with SBP according to the current guidelines (polymorphonuclear (PMN) cell count > 250 mm^3^) as compared to patients who did not have SBP. This result is agreement with those reported by Burri et al. [19], Fernandes et al. [17], and Abdel-Razzik et al. [15]. A significant positive correlation was observed between ascitic fluid calprotectin and ascitic fluid protein and PMN cell count [20]. Burri et al. [19] reported that ascitic calprotectin levels correlated well and reliably with PMN count. Samples with PMN > 250/mm^3^ also had higher ascitic calprotectin levels than the samples with PMN ≤ 250/mm. Although ascitic fluid Calprotectin was higher in Child–Pugh A, B vs. C (31.2 ± 52.5 vs. 23.3 ± 12.9 respectively), MELD SCORE < 11 VS. MELD SCORE > 11 (42.9 ± 67.2 vs. 18.9 ± 10.2 respectively), it did not reach statistical significance, leading us to assume that is do not have a potential prognostic value. Similar results of the absent of association between ascitic calprotectin and Child–Pugh and MELD scores were reported by a previous study by Lutz et al. [14] and by Weil et al. [18]. However, the small cohort prevent us from drawing clear conclusions.

There are several limitations to the current study that merit consideration. First, we included all patients with ascites, irrespective of the etiology, and it may be that our results cannot be popular to all patients with liver cirrhosis. Second, our sample size was comparatively small, and we will need larger studies to evaluate this test in different clinical settings and to establish a reliable cutoff for ascitic calprotectin for optimal identification of PMN counts > 250/mm^3^. Another limitation is that the rate of SBP in our study was 25.8%, while the reported rate in the literature is about 10% [21], this is probably due to the inclusion of patients who referred because of suspicion of SBP and paracentesis of tense ascites, which are from the beginning have a high probability of SBP.

In conclusion: ascitic calprotectin appears to be a reliable method for diagnosing SBP in patients with liver cirrhosis. It may present an alternative to other conventional diagnostic methods. In one hand there is a correlation between the level of calprotectin and the diagnosis of SBP. In the other hand the study pointing to the absence of the prognostic value of ascetic calprotectin is staging disease severity.

## Data Availability

The data are found at the gastroenterology department at Galilee Medical Center, Nahariya, Israel, and will be available upon reasonable request.

## Abbreviations

SBP: Spontaneous bacterial peritonitis
PMN: Polymorphonuclear
MELD: Model for end-stage liver disease
AST: Aspartate aminotransferase
ALT: Alanine aminotransferase
ALK: Alkaline phosphatase
ELISA: Enzyme-linked immunosorbent assay
SD: Standard deviation
NASH: Non-alcoholic steatohepatitis
ROC: Receiver operating characteristic curve
